# Abiraterone acetate after progression with enzalutamide in chemotherapy-naïve patients with metastatic castration-resistant prostate cancer: a multi-center retrospective analysis

**DOI:** 10.1186/s13104-016-2279-9

**Published:** 2016-10-18

**Authors:** Yoko Yamada, Nobuaki Matsubara, Ken-ichi Tabata, Takefumi Satoh, Naoto Kamiya, Hiroyoshi Suzuki, Takashi Kawahara, Hiroji Uemura, Akihiro Yano, Satoru Kawakami

**Affiliations:** 1 Department of Breast and Medical Oncology, National Cancer Center Hospital East, 6-5-1 Kashiwanoha, Kashiwa, Chiba, 277-8577 Japan; 2 Department of Urology, Kitasato University School of Medicine, Sagamihara-shi, Kanagawa Japan; 3 Department of Urology, Toho University Sakura Medical Center, Chiba, Japan; 4 Department of Urology, Yokohama City University Graduate School of Medicine, Yokohama, Kanagawa Japan; 5 Department of Urology, Saitama Medical Center, Saitama Medical University, Saitama, Japan

**Keywords:** Metastatic castration-resistant prostate cancer, Abiraterone acetate, Enzalutamide, PSA, Cross-resistance

## Abstract

**Background:**

Both abiraterone acetate (AA) and enzalutamide are promising agents for patients with pre- and post-chemotherapy metastatic castration-resistant prostate cancer (mCRPC). Several retrospective analysis suggested clinical cross-resistance between these agents in patients previously treated with docetaxel. However, data on the antitumor activity of AA as a second androgen receptor-targeting new agent after the failure of enzalutamide in chemotherapy-naive mCRPC patients is unavailable.

**Methods:**

Patients with chemotherapy-naïve mCRPC who were treated with AA after disease progression with enzalutamide, were retrospectively reviewed at five institutions. Primary outcome measure was the rate of any prostate-specific antigen (PSA) decline. Secondary outcome measures were progression-free survival (PFS) and overall survival (OS) with subsequent AA treatment. We also performed correlation analysis between previous PSA response, PFS duration to enzalutamide and subsequent PSA response, PFS duration to AA.

**Results:**

A total of 14 patients were identified. Any PSA declines and PSA decline ≥50 % with AA treatment, were observed in 36 and 7 % of patients, respectively. Median PFS with initial enzalutamide was 5.0 months (95 % CI 3.7–6.4 months), and for subsequent AA treatment was 3.4 months (95 % CI 0.8–6.0 months). Median OS from initiation of AA was 9.1 months (95 % CI 5.6–12.5 months). No significant correlations were observed between these PSA responses (Pearson r = −0.67, p = 0.82) and PFS duration (Kendall tau r = 0.33, p = 0.87).

**Conclusions:**

The PSA decline with subsequent AA treatment in chemotherapy-naive mCRPC patients after a failure of enzalutamide was modest, however, the PFS and OS with subsequent AA treatment were comparable to those of enzalutamide previously reported as a second androgen receptor-targeting new agent after AA failure. The PSA response and PFS duration to previous enzalutamide treatment did not predict those of subsequent AA treatment.

## Background

Prostate cancer, as the second most common male cancer worldwide [1], and the third most common cause of male cancer deaths in developed countries, is a major health concern [2]. These trends are no exception in Japan, where the number of prostate cancer patients has been rapidly increasing. Recently, the Cancer Information Service of the National Cancer Center of Japan, indicated that prostate cancer was projected to become the most common cancer, and the cause of a sixth of cancer deaths among men in Japan in 2015 [3].

Prostate cancer is initially an androgen-dependent disease and responds well to androgen-deprivation treatment (ADT) [4]. However, almost all patients, unfortunately, experience disease progression during ADT within several years, despite attaining a castrate levels of testosterone, at which point they are described as having metastatic castration-resistant prostate cancer (mCRPC) [5]. After developing mCRPC, this disease state is considered incurable and life-threatening [6].

Until recently, docetaxel was the only approved agent that improved overall survival in mCRPC patients. However, several relatively new agents have induced promising improvements in overall survival in patients with mCRPC, and have, consequently, been introduced into daily clinical practice. Of these new agents, abiraterone acetate (AA) [7, 8] and enzalutamide [9, 10] are oral agents whose mechanism of action is through an androgen receptor (AR) signaling pathway. AA and enzalutamide have already been approved for mCRPC patients, regardless of prior docetaxel treatment, based on positive results from a large randomized phase 3 trial. The success of new agents that target the AR means that the AR signaling pathway remains an important driver of prostate cancer in the castration-resistant state [11]. Both AA and enzalutamide are increasingly being used in chemotherapy-naïve patients with mCRPC for their efficacy, as well as for their, favorable toxicity profiles.

In spite of the rapid introduction of AA and enzalutamide into daily practice, several clinical questions concerning new AR-target agents remain unanswered. A major clinical question is whether another subsequent AR-targeting agent will still retain antitumor activity after becoming AR-targeted agent resistant. Several small retrospective analyses reported on the efficacy of enzalutamide in mCRPC patients after progressing on AA. However, almost all of these analyses were restricted to patients who had already been treated with docetaxel [12–15], and only one small study investigated chemotherapy-naïve patients [16]. In addition, treatment in the reverse sequence, enzalutamide followed by AA, has been reported in only patients who had already been treated with docetaxel [17, 18]. Based on these results of sequential treatment with new AR-targeting agents, the efficacy of a second AR-targeting agent was modest, with median time to progression of approximately 3–4 months.

To the best of our knowledge, published data on chemotherapy-naïve mCRPC patients treated with enzalutamide and followed by AA have not been reported as yet. We assume the antitumor activity of AA treatment in chemotherapy-naïve mCRPC patients after progressing with enzalutamide might also be modest. The objectives of the current retrospective analysis, therefore, were to reveal the efficacy and clinical outcome of AA treatment in chemotherapy-naïve mCRPC patients who had previously undergone treatment with enzalutamide. In order to investigate this clinical question, we conducted a multi-institutional retrospective analysis.

## Methods

### Patients

We conducted a multicenter retrospective study which was performed in five institutions (National Cancer Center Hospital East, Yokohama City University Hospital, Kitasato University Hospital, Toho University Sakura Medical Center and Saitama Medical Center Saitama Medical University). This study was carried out in accordance with the Declaration of Helsinki and Japanese ethical guidelines for epidemiological research. We obtained institutional review board waivers from all participating institutional review board chairpersons to conduct this study.

Chemotherapy-naïve patients with mCRPC who had been treated with enzalutamide until the time of disease progression, and who were subsequently treated with AA, were eligible for this analysis. CRPC patients were defined based on evidence of disease progression (clinical, radiographic or prostate-specific antigen (PSA) elevation) despite castrate serum testosterone levels and continuous luteinizing hormone-releasing hormone analogues/antagonist treatment. Patients with non-metastatic CRPC, or who were treated with docetaxel before the initiation of enzalutamide, were excluded in this analysis. Treatment with enzalutamide continued until the time of disease progression according to the Prostate Cancer Working Group 2 criteria [19]. Patients who discontinued treatment with enzalutamide due to unacceptable toxicity were excluded from this analysis. This retrospective study investigated the direct anti-tumor activity of AA treatment in chemotherapy-naïve mCRPC patients who were resistant to enzalutamide treatment. Therefore, in this analysis, all patients experienced disease progression with enzalutamide.

Between the termination of enzalutamide, and the initiation of AA treatment, patients treated with any vintage hormonal manipulations, such as first-generation anti-androgen receptor inhibitor (flutamide, bicaltamide), steroid (dexamethasone, prednisolone) and estrogen agent, were allowed into this study, but if treated with any chemotherapy or investigational drugs, patients were excluded.

All data of patient characteristics and treatment outcomes with enzalutamide and AA were collected retrospectively from medical records of individual institutions. Information on the following parameters were made available for all patients: age, Gleason score, prior treatment history with vintage hormonal manipulations, serum PSA at the time of baseline enzalutamide and AA initiation, number and sites of metastasis, Eastern Cooperative Oncology Group (ECOG) performance status (PS), serum PSA level during treatment, treatment duration with enzalutamide and AA, type of disease progression with enzalutamide and AA, and survival status.

### Outcomes measurement and statistics

The primary outcome measure of this investigation was to investigate the frequency of any PSA decline from baseline. Any PSA decline was defined as a PSA decrease from baseline, regardless of degree of decrease during subsequent AA treatment. The secondary outcome measures were PFS and overall survival (OS) with subsequent AA treatment. PFS was defined as the time from the initiation of AA treatment to PSA progression or radiographic progression according to PCWG2 criteria [19], or clinical progression. OS was defined as the time from initiation of AA to death from any cause or censoring on 30 November 2015. Kaplan–Meier estimates were used for PFS and OS. A correlation analysis between factors were evaluated using Kendall tau or Pearson correlation test, where appropriate. All tests were two-sided and considered significant at p < 0.05. All statistical analyses were performed using SPSS 22.0 statistical package for Windows (SPSS, IBM, Chicago, IL., USA).

## Results

### Patient characteristics and outcomes with previous enzalutamide treatment

**Fig. 1:**
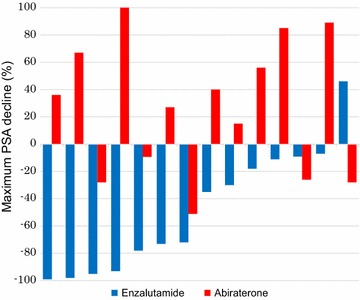
Waterfall plot showing maximum PSA reduction of prior enzalutamide and subsequent abiraterone acetate treatment in each patient

A total of 14 patients, who experienced disease progression with enzalutamide treatment and were subsequently treated with AA, were eligible for this analysis. Patients and disease characteristics at baseline and at the time of initiation of enzalutamide treatment are shown in Table 1. Patients who had previously received a radical prostatectomy or radial radiation therapy made up only 21 % of the total. The median age, at the time of the first enzalutamide dose, was 78 years. All patient had been treated with vintage hormonal manipulations, such as first-generation anti-androgen receptor inhibitors, steroids and estrogen agents prior to the initiation of the enzalutamide treatment. The median number of treatment line with vintage hormonal manipulations prior to the initiation of enzalutamide treatment was three lines, not including luteinizing hormone-releasing hormone agonist/antagonist. The median interval between the development of mCRPC to the initiation of enzalutamide treatment was 5.1 months. At the time of initiation of enzalutamide, almost all patients (79 %) were in good general condition, with an ECOG PS of 0 or 1, and only one patient had a visceral metastasis. However, all patients displayed bone metastasis, and nearly half (43 %) had a huge bone metastasis spreading as an extent of disease (EOD) score of 3. The outcomes with previous enzalutamide treatment are summarized in Table 2. During the first round enzalutamide treatment, PSA declines of ≥30 and ≥50 % were observed in 64 and 50 % of patients, respectively. Almost of all patients (93 %) achieved some PSA decline, regardless of degree. A waterfall plot figure of the maximal PSA decline with enzalutamide treatment is presented in Fig. 1. The types of disease progression were PSA progression of disease (PD) in 72 %, radiographic PD in 21 % and clinical PD in 7 % of patients. The median PFS for patients treated with enzalutamide was 5.0 months (95 % CI 3.7–6.4 months).

**Table 1:** Patient characteristics (n = 14)

Baseline characteristics	
Gleason score, n (%)	
≤7	1 (7)
8	6 (43)
9	6 (43)
10	1 (7)
Prior local treatment, n (%)	3 (21)
Radical prostatectomy, n	1
Radical radiation therapy, n	2
Patient characteristics at initiation of enzalutamide	
Median age, years (range)	78 (50–88)
Median time from CRPC to initiation of enzalutamide, mo (range)	5.1 (1.3–75.4)
ECOG PS, n (%)	
0	5 (36)
1	6 (43)
2	3 (21)
≥3	0
Number of previous vintage hormone manipulations, median (range)	3 (2–7)
Metastatic site, n (%)	
Bone	13 (100)
EOD1	1 (7)
EOD2	7 (50)
EOD3	6 (43)
EOD4	0
Lymph node	6 (43)
Lung	1 (7)
Liver	1 (7)
PSA (ng/ml), median (range)	89.9 (22.4–445.6)
Hemoglobin (g/l), median (range)	11.4 (9.7–14.0)
LDH (U/l), median (range)	254 (173–2028)
ALP (U/l), median (range)	205 (99–1303)
Patient characteristics at time of initiation of abiraterone acetate	
Median time from enzalutamide discontinuation to initiation of abiraterone acetate, day (range)	1 (1–69)
ECOG PS, n (%)	
0	3 (21)
1	5 (36)
2	6 (43)
≥3	0
PSA (ng/ml), median (range)	38.0 (8.6–572.1)
Hemoglobin (g/l), median (range)	11.8 (9.2–14.0)
LDH (U/l), median (range)	202 (143–960)
ALP (U/l), median (range)	316 (117–717)

**Table 2:** Treatment outcome of prior enzalutamide and subsequent abiraterone acetate treatment

	Enzalutamide (n = 14) n (%)	Abiraterone (n = 14) n (%)
Any PSA decline	13 (93)	5 (36)
PSA decline ≥30 %	9 (64)	1 (7)
PSA decline ≥50 %	7 (50)	1 (7)
Median PFS, mo (95 % CI)	5.0 (3.7–6.4)	3.4 (0.8–6.0)
Type of progression		
PSA PD	10 (72)	8 (57)
Radiographic PD	3 (21)	5 (36)
Clinical PD	1 (7)	1 (7)

### Antitumor activity with subsequent AA treatment in patients with enzalutamide resistance

**Fig. 2:**
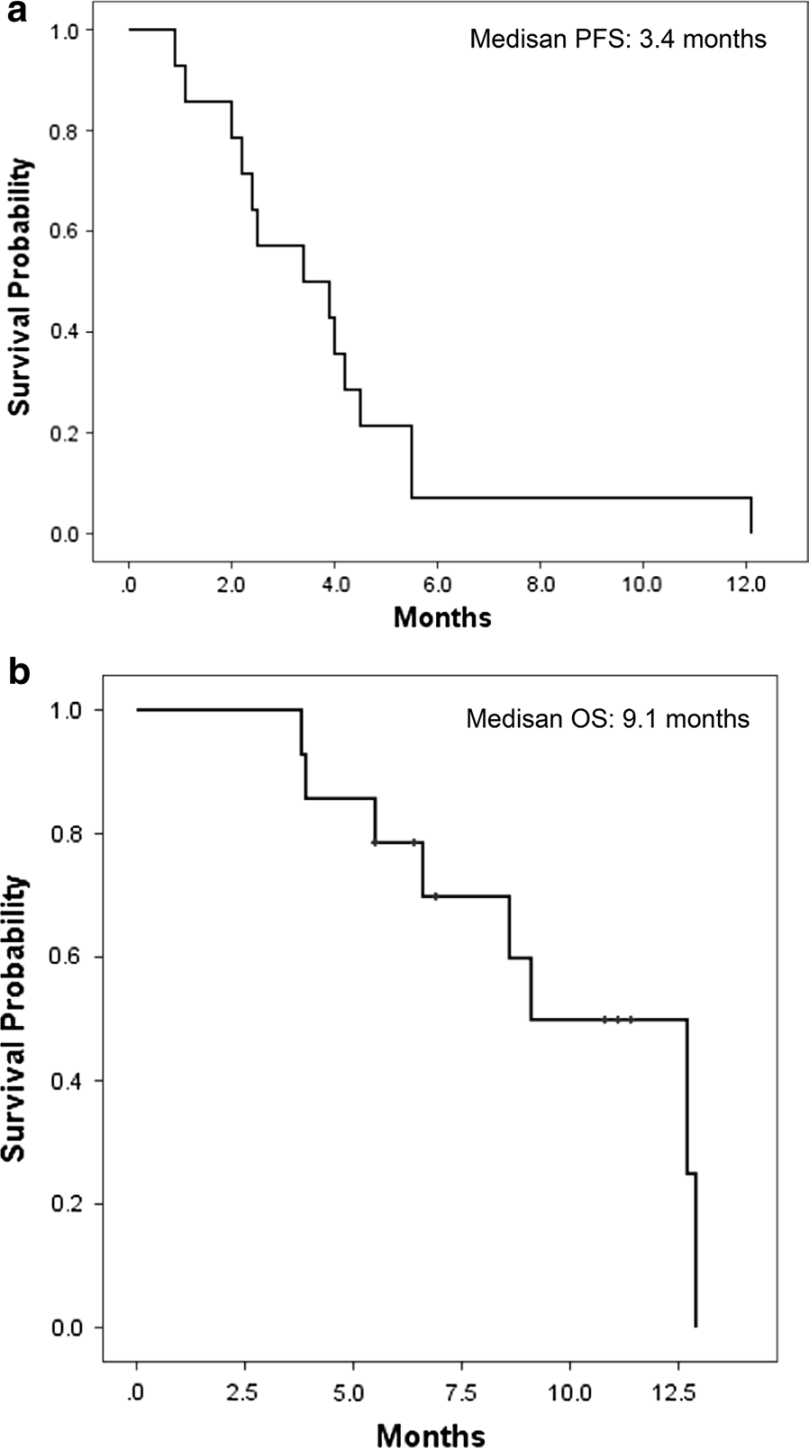
Kaplan–Meier curve of **a** progression-free survival with subsequent abiraterone acetate treatment **b** overall survival from initiation of abiraterone acetate treatment

The median interval between the last dose of enzalutamide and the initiation of AA was 1 day (range 1–69 days). Only two patients (14 %) received a systemic treatment with another vintage hormonal manipulation (flutamide, dexamethasone) between the cessation of enzalutamide treatment and the start of AA treatment. Patients and disease characteristics at the time of initiation of AA treatment are shown in Table 1. Baseline characteristics, such as ECOG PS, and laboratory data, including serum PSA levels at the initiation of AA treatment, were similar to those at the initiation of enzalutamide treatment. All patients were started with a standard dose and schedule of AA, orally 1000 mg, once daily, co-administered with 5 mg prednisone bid. A PSA decline, regardless of the degree of decline, was observed in 36 % of patients. However, PSA declines ≥30 and ≥50 % were observed in only 7 and 7 % of patients, respectively. A waterfall plot figure of maximal PSA decline with subsequent AA treatment is also presented in Fig. 1. At the time of the censoring date, all patients had discontinued AA treatment due to disease progression. The type of disease progression was PSA PD in 57 %, radiographic PD in 36 % and clinical PD in 7 % of patients. No patient discontinued AA treatment due to unacceptable toxicity. The median PFS for patients treated with AA was 3.4 months (95 % CI 0.8–6.0 months), as shown in the Kaplan–Meier Fig. 2a. Until the censoring date, 8 of 14 patients (57 %) died, and all causes of death were due to mCRPC. The median OS from initiation of AA was 9.1 months (95 % CI 5.6–12.5 months), as shown in the Kaplan–Meier Fig. 2b.

We performed a correlation analysis between the PSA responses, PFS duration to prior enzalutamide and these to subsequent AA treatments. No significant correlations were observed between these PSA responses (Pearson r = −0.67, p = 0.82, figure not shown) and PFS duration (Kendall tau r = 0.33, p = 0.87, Figure not shown).

## Discussion

In this retrospective analysis, we revealed the efficacy of AA treatment after enzalutamide failure in chemotherapy-naïve mCRPC patients. To the best of our knowledge, this is the first investigation to examine the efficacy of AA as a second AR-targeting agent after enzalutamide, but before the initiation of docetaxel treatment. Presently, this treatment sequence, enzalutamide followed by AA, may be more popular than in reverse in Japan since the timing of approval for the use of enzalutamide and AA in Japan was in reverse order to that of the US and EU. In other words, in Japan, enzalutamide was approved prior to the approval of AA for mCRPC patients.

We found the treatment with AA after enzalutamide failure in chemotherapy-naïve patients with mCRPC showed limited activity. Any PSA decline, or a PSA decline ≥30 or ≥50 % were observed in 36, 7 and 7 % of patients, respectively. In addition, the median PFS was 3.4 months (95 % CI 0.8–6.0 months). The anti-tumor activity of AA was inferior to that reported in clinical trials of patients that were chemotherapy- and enzalutamide-naïve, such as, COU-AA-302 [8] and JPN-201 [20]. There are several conceivable reasons for this reduced efficacy of AA. Firstly, the disease burden and patient characteristics of our cohort may have been worse than those of the above registered clinical trials. A recent retrospective analysis from the UK revealed that metastatic spread is an independent predictive factor to a PSA response to AA treatment [21]. In fact, the number of bone metastases with an EOD score at the time of initiation of AA treatment in the present study seems to indicate a greater disease burden compared with those of published data of registration trials.

Another plausible reason is the potential of cross-resistance between enzalutamide and AA, in other words, the existence of an overlapping resistance mechanism. A target common to both enzalutamide and AA is the AR-signaling pathway, however these differ in their mechanisms of activity, with enzalutamide inhibiting AR directly and AA inhibiting extra-gonadal and intra-tumor androgen synthesis. The Specific mechanism of resistance to enzalutamide and AA has not yet been clearly identified. However, a recently published paper reported that the androgen receptor splice variant 7 (AR-V7) may have the potential to be a reliable predictive biomarker for AA and enzalutamide [22], in addition, a prior treatment history with enzalutamide or AA was associated with AR-V7 positivity. These data suggest the resistance mechanisms for AA and enzalutamide may be overlap. However, this seems implausible given that 36 % of patients in our study achieved a PSA decline with AA after enzalutamide failure. In addition, one patient was not observed to show any PSA decline with prior enzalutamide treatment, but achieved a PSA decline with subsequent AA treatment. Our results, therefore, suggest that there may be different and non-overlapping mechanisms of resistance for AA and enzalutamide.

The median PFS for prior enzalutamide treatment in chemotherapy- and AA-naïve patients of the present study was 5.0 months, which is shorter than those from previous studies. A subgroup analysis of Japanese patients from the PREVAIL phase 3 trial reported a median PFS for Japanese patients of 3.7 months [23], which is comparable to the median PFS of the present study. Based on these data, including ours, enzalutamide activity in Japanese patients seems to be inferior to its activity in non-Japanese patients. A conceivable reason for differences may depend on differences in treatment histories with alternate hormonal therapies before the initiation of enzalutamide. In Japanese daily practice, almost all patients receive maximum androgen blockade as an initial ADT. In the case of the failure of a maximum androgen blockade, Japanese patients are generally treated with subsequent second- and third-line hormonal treatments using vintage hormonal manipulations. We postulate that such an intense treatment history using vintage hormonal manipulations prior to the initiation of enzalutamide/AA treatment in Japanese patients affected the reduced anti-tumor activity seen with enzalutamide AA treatment.

In contrast, the median PFS and OS with subsequent AA treatment in the present study was 3.4 and 9.1 months, which is comparable to the PFS and OS of enzalutamide previously reported as a second AR-targeting agent after AA failure. A recent published retrospective analysis based on US claims database indicates that only 30–40 % of mCRPC patients can receive docetaxel treatment in the real world [24]. Collectively, our results and real world data suggest that AA followed by enzalutamide, or the reverse sequence, has the potential to be an alternative treatment option for patients unfit for docetaxel based chemotherapy. However, in order to be a reliable treatment option, larger prospective studies need to be conducted for validation.

The present investigation also revealed another important finding that the outcomes, such as PSA changed and PFS duration with previous enzalutamide treatment could not predict subsequent outcomes with AA treatment. From the correlation analysis of present study, no significant correlations were observed in not only the PSA responses but also PFS durations. These results from our investigation suggest that the PSA response and PFS duration to a prior enzalutamide treatment might not be useful to selecting patients for subsequent treatment.

Several potential limitations exist in the present study. Firstly the cohort used was small with only 14 patients, and therefore, our analysis may be potentially underpowered. Secondly, the present investigation include only patients who had short PFS with enzalutamide treatment, thus, our results might not be reflective of the general mCRPC patients. Thirdly, this is a retrospectively designed study. Finally, the timing of the initiation of AA treatment, and definition of disease progression were not uniform, but determined on individual physicians. Thus, scanning intervals and scanning devices during enzalutamide and AA treatment varied among patients. However, these procedures were similar to those of real-world clinical practice. Therefore, we assume that the results from present study will become useful references in daily clinical practice, especially for patients who do not have a suitable general condition for docetaxel based chemotherapy initiation.

## Conclusions

Our investigation revealed that the PSA response to subsequent AA treatment in chemotherapy-naïve, and enzalutamide refractory mCRPC patients was modest. However, the PFS and OS was comparable to those of enzalutamide previously reported as a second AR-targeting agent after AA failure. The PSA response and PFS duration to previous enzalutamide treatment couldn`t predict the efficacy of subsequent AA treatment. These findings require validation in a larger prospective trial.

## Abbreviations

AA: abiraterone acetate
ADT: androgen-deprivation treatment
AR: androgen receptor
AR-V7: androgen receptor splice variant
ECOG: Eastern Cooperative Oncology Group
EOD: extent of disease
mCRPC: metastatic castration-resistant prostate cancer
OS: overall survival
PD: progression of disease
PFS: progression-free survival
PSA: prostate-specific antigen
